# Supplementary figures and images for: NFATc1 marks articular cartilage progenitors and negatively determines articular chondrocyte differentiation
Source: eLife. 2023 Feb 15;12:e81569. doi: 10.7554/eLife.81569 (PMC10076019; doi:10.7554/eLife.81569)

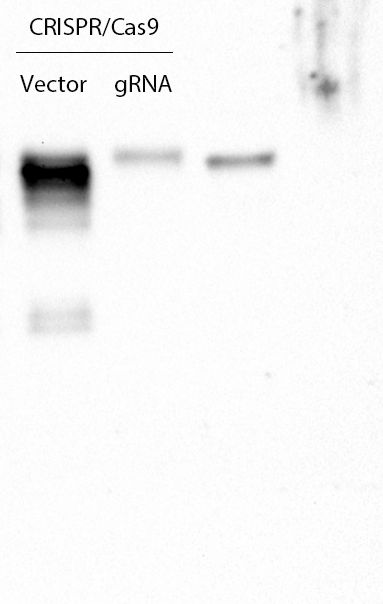

Supplement: Figure 5—figure supplement 1—source data 1. [file elife-81569-fig5-figsupp1-data1.zip › Fig. S5-source data/NFATc1-CRISPR.tif]

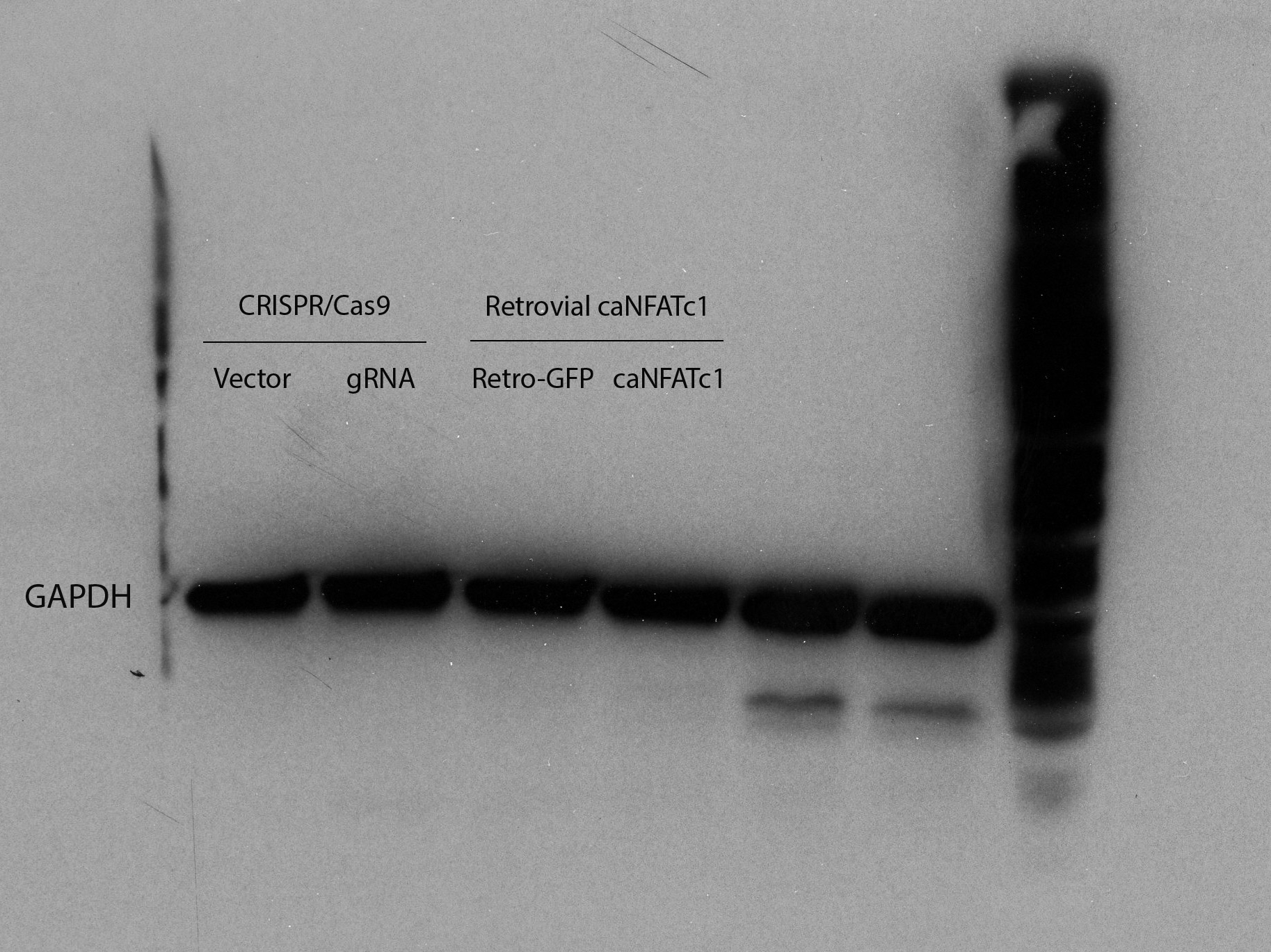

Supplement: Figure 5—figure supplement 1—source data 1. [file elife-81569-fig5-figsupp1-data1.zip › Fig. S5-source data/GAPDH.tif]

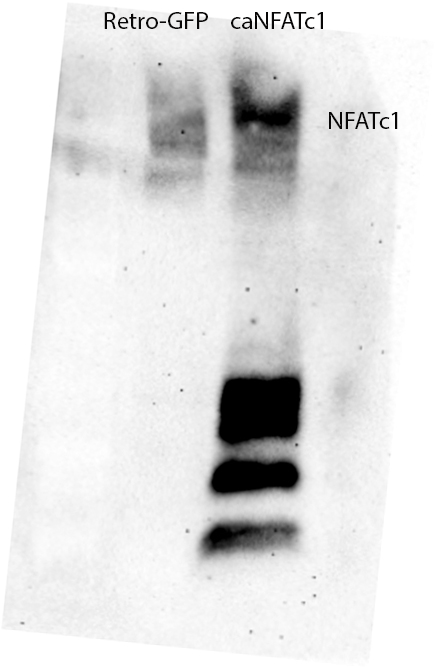

Supplement: Figure 5—figure supplement 1—source data 1. [file elife-81569-fig5-figsupp1-data1.zip › Fig. S5-source data/NFATc1-retroviral caNFATc1.tif]
